# Targeted Exome Sequencing of Genes Involved in Rare CNVs in Early-Onset Severe Obesity

**DOI:** 10.3389/fgene.2022.839349

**Published:** 2022-03-07

**Authors:** Petra Loid, Minna Pekkinen, Taina Mustila, Päivi Tossavainen, Heli Viljakainen, Anna Lindstrand, Outi Mäkitie

**Affiliations:** ^1^ Children's Hospital, University of Helsinki and Helsinki University Hospital, Helsinki, Finland; ^2^ Folkhälsan Research Center, Genetics Research Program, Helsinki, Finland; ^3^ Research Program for Clinical and Molecular Metabolism, Faculty of Medicine, University of Helsinki, Helsinki, Finland; ^4^ City of Turku Wellfare Services, Diabetes Care, Turku, Finland; ^5^ Department of Pediatrics, PEDEGO Research Unit, Medical Research Center, Oulu University Hospital, University of Oulu, Oulu, Finland; ^6^ Faculty of Medicine, University of Helsinki, Helsinki, Finland; ^7^ Department of Molecular Medicine and Surgery, Center for Molecular Medicine, Karolinska Institutet, Stockholm, Sweden; ^8^ Department of Clinical Genetics, Karolinska University Hospital, Stockholm, Sweden

**Keywords:** childhood obesity, gene panel, rare variants, candidate genes, copy number variants

## Abstract

**Context:** Rare copy number variants (CNVs) have been associated with the development of severe obesity. However, the potential disease-causing contribution of individual genes within the region of CNVs is often not known.

**Objective:** Screening of rare variants in genes involved in CNVs in Finnish patients with severe early-onset obesity to find candidate genes linked to severe obesity.

**Methods:** Custom-made targeted exome sequencing panel to search for rare (minor allele frequency <0.1%) variants in genes affected by previously identified CNVs in 92 subjects (median age 14 years) with early-onset severe obesity (median body mass index (BMI) Z-score + 4.0).

**Results:** We identified thirteen rare heterozygous variants of unknown significance in eleven subjects in twelve of the CNV genes. Two rare missense variants (p.Pro405Arg and p.Tyr232Cys) were found in *SORCS1*, a gene highly expressed in the brain and previously linked to diabetes risk. Four rare variants were in genes in the proximal 16p11.2 region (a frameshift variant in *TAOK2* and missense variants in *SEZ6L2*, *ALDOA* and *KIF22*) and three rare missense variants were in genes in the 22q11.21 region (*AIFM3, ARVCF* and *KLHL22*).

**Conclusion:** We report several rare variants in CNV genes in subjects with childhood obesity. However, the role of the individual genes in the previously identified rare CNVs to development of obesity remains uncertain. More studies are needed to understand the potential role of the specific genes within obesity associated CNVs.

## Introduction

Copy number variants (CNVs) are structural variants that include duplications or deletions of genomic regions ranging from 50 bp to several megabases (Zarrei et al., 2015). These structural variants can arise by different mutational mechanisms such as replication, repair of double-strand breaks and recombination (Gu et al., 2008; Carvalho and Lupski, 2016). CNVs contribute to human diversity but also to several diseases, including neurodevelopmental disorders and severe obesity (Wang et al., 2010; Wheeler et al., 2013; D'Angelo et al., 2018; Saeed et al., 2020).

Deletion in 16p11.2 region is the most established CNV linked to early-onset severe obesity (Bochukova et al., 2010; Walters et al., 2010). CNVs also contribute to syndromic obesity (Vuillaume et al., 2014; Pettersson et al., 2017; D’Angelo et al., 2018; Selvanayagam et al., 2018; Saeed et al., 2020; Madeo et al., 2021). The identification of rare CNVs in obesity has led to the discovery of several novel candidate genes. However, in many cases the disease-causing gene within the region of CNVs is unclear. Indeed, unraveling the pathogenicity of CNVs remains a major challenge (Nowakowska, 2017). Pathogenic CNVs are usually large and contain genes with high evolutionary copy number conservation. It has been proposed that dosage sensitivity of individual genes within the region of CNVs is a frequent cause of CNV pathogenicity. Haploinsufficiency is commonly used as a model of gene dosage sensitivity and predictions of haploinsufficiency have been used to predict the impact of CNVs (Huang et al., 2010; Lek et al., 2016; Rice and McLysaght, 2017).

We previously identified rare CNVs enriched in subjects with severe childhood obesity (Pettersson et al., 2017). In a case-control study of 90 obese subjects and 67 normal-weight controls, using array comparative genomic hybridization, we identified rare CNVs in 17 obese subjects. However, the pathogenicity of several of the identified CNVs remained unclear and the potential phenotypic contribution of the genes within the CNVs is not known. We hypothesized that rare variants in the genes within the CNVs might contribute to the development of obesity. We designed a custom-made targeted exome sequencing panel, including all genes located in the regions of the rare CNVs identified in our cohort of patients with childhood obesity, in search for rare variants and novel candidate genes.

## Methods

### Study Subjects

This study included 92 children and adolescents with severe early-onset obesity, defined as height-adjusted weight >60% before 10 years of age according to Finnish growth standards (Saari et al., 2011). The study subjects were recruited through pediatric endocrine clinics at Helsinki University Hospital, Seinäjoki Central Hospital and Oulu University Hospital during years 2011–2017. We have previously investigated the study subjects by targeted exome sequencing for rare variants in 24 genes related to the leptin-melanocortin pathway or hypothalamic development (Loid et al., 2020). Altogether 80% of the patients in this study have also been screened for rare CNVs by array comparative genomic hybridization (a-CGH). In our previous study we identified rare CNVs in 17 obese subjects (Pettersson et al., 2017) and 14 of these 17 subjects with rare CNVs participated in the present study. The study was approved by Research Ethics Committees of Hospital District of Helsinki and Uusimaa, the Pirkanmaa Hospital District and the Northern Ostrobothnia Hospital District. Informed written consents were obtained from all the participants or their parents/guardians. Clinical and growth data were collected from hospital records and anthropometric measurements were obtained during a study visit. Age-and gender specific BMI Z-scores were derived based on the World Health Organization reference values (www.who.int/childgrowth/standards).

### Targeted Exome Sequencing

We designed the probes for targeted exome sequencing using SeqCap EZ Choice Library and Nimble Design (Roche NimbleGen, United States). DNA capture and sequencing were performed at Oxford Genomics Centre. Sequence reads were aligned to reference genome GRCh37. In this study, we included 117 genes involved in previously identified rare CNVs (Pettersson et al., 2017). The genes are listed in Supplementary Table S1. We filtered variants using Varaft (version 2.17) (Desvignes et al., 2018). We selected coding and splice site variants with an allele frequency of <0.001 in the Genome Aggregation Database (gnomAD) database (http://gnomad.broadinstitute.org), the 1000 Genomes Project (http://www.internationalgenome.org), and the Sequencing Initiative Suomi database (SISu) (http://www.sisuproject.fi). In a second analysis, we also evaluated coding and splice site variants with an allele frequency of <0.005 to explore whether change of allele frequency threshold would add any potentially interesting discoveries, considering that severe obesity is not an extremely rare phenotype. *In silico* predictions were performed using Sorting Intolerant From Tolerant (SIFT) (Kumar P. et al., 2009), Polymorphism Phenotyping version 2 (PolyPhen2) (http://genetics.bwh.harvard.edu/pph2/), MutationTaster2 (Schwarz et al., 2014), Combined Annotation Dependent Depletion (CADD) scores (https://cadd.gs.washington.edu/), Mendelian Clinically Applicable Pathogenicity (M-CAP) (http://bejerano.stanford.edu/mcap/), Rare Exome Variant Ensemble Learner (REVEL) score (Ioannidis et al., 2016) and splice AI (https://spliceailookup.broadinstitute.org). We selected rare variants predicted to be pathogenic by several of the abovementioned prediction tools including a CADD score >20. Integrative Genomics Viewer (IGV) was used for visualization of the variants. The identified variants were classified according to the American Collage of Medical Genetics (ACMG) guidelines (Richards et al., 2015) using VarSome tool (Kopanos et al., 2019). Parental and siblings’ samples was obtained from two available families with the identified rare variants and Sanger sequencing was performed to determine inheritance pattern.

## Results

### Characteristics of Study Subjects

The cohort in this study comprised 92 subjects (51% males) with median age of 13.7 years (interquartile range (IQR) 10.6–16.8 years) and median BMI Z-score of +4.0 (IQR + 3.4 to + 4.9). The study subjects fulfilled our inclusion criteria (height-adjusted weight >60%) at median age of 6.0 years (IQR 4.5–7.0 years). Of the 68 school-aged study subjects for whom information on learning difficulties was available, ten (15%) had learning difficulties. Two study subjects had developmental delay based on previous clinical investigations. In these two patients Prader-Willi syndrome had been ruled out. Ten participants had insulin resistance based on oral glucose tolerance test.

### Targeted Exome Sequencing

**FIGURE 1:**
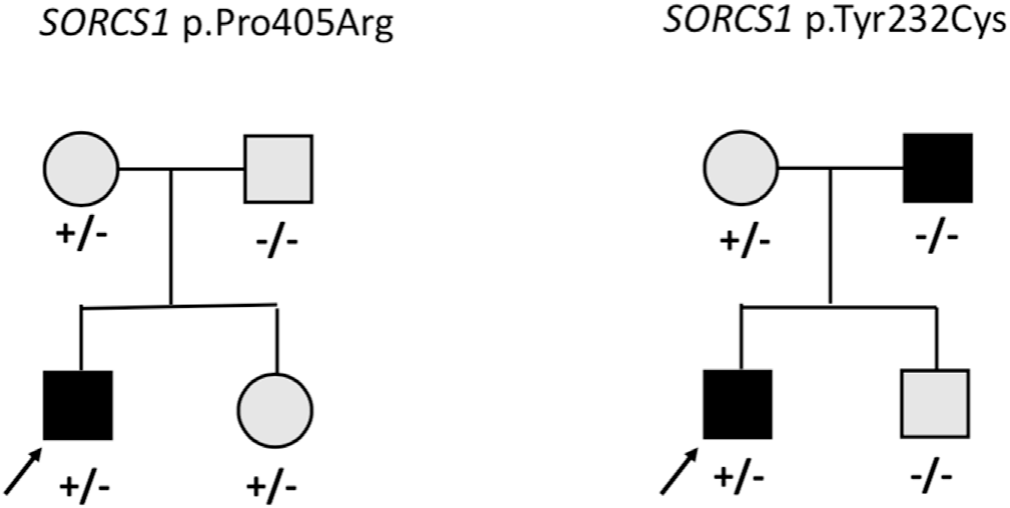
Pedigrees of the families with missense variants in *SORCS1*. Square = male, circle = female, +/− heterozygote, −/− wildtype. Black square/circle = obesity, gray square/circle = overweight.

On average 99.3% of the bases in the coding exons were covered by at least 100 reads and the mean read depth of coverage was 750X. We identified 13 rare heterozygous variants in 11 subjects with early-onset obesity. Four of them had learning difficulties and one had developmental delay with autistic features. The variants with allele frequency of <0.001 were in 12 of the genes in the panel, namely *AIFM3*, *ALDOA*, *ARVCF*, *BRAF*, *CHD1L*, *KIF22, KLHL22*, *MCTP2*, *SEZ6L2*, *SORCS1*, *TAOK2* and *XRN1*. Table 1 presents the rare variants with allele frequencies in gnomAD and *in silico* prediction values. For the rare splicing variant in *CHDL1* we also assessed the SpliceAI score, which was 0.990 (donor loss). In one of the genes (*SORCS1*) we identified two rare variants; p.Pro405Arg and p.Tyr232Cys. Figure 1 presents the segregation of the *SORCS1* variants in the two families. In both families the variant was inherited from a parent with overweight. However, also other family members without the variant were overweight or obese.

**TABLE 1:** The identified rare variants with minor allele frequencies (MAF) in gnomAD and *in silico* prediction values.

Study subject	1	2	3			4	5	6	7	8	9		10	11
BMI (Z-score)	+5.8	+5.1	+4.0			+4.3	+2.8	+2.9	+3.8	+4.5	+3.7		+5.0	+3.8
Associated features	—	insulin resistance, learning difficulties	learning difficulties			—	—	—	learning difficulties	developmental delay, autistic features	learning difficulties		—	—
Gene	*CHD1L*	*KIF22*	*SORCS1*	*SEZ6L2*		*TAOK2*	*BRAF*	*ALDOA*	*ARVCF*	*KLHL22*	*SORCS1*	*AIFM3*	*MCTP2*	*XRN1*
Type of variant	Splicing	Missense	Missense	Missense		Frameshift deletion	Missense	Missense	Missense	Missense	Missense	Missense	Missense	Missense
Genotype	Het	Het	Het	Het		Het	Het	Het	Het	Het	Het	Het	Het	Het
Chromosome position (hg19)	1:146756173G > A	16:29810594C > T	10:108466322G > C	16:29899049G > A		16:29998896 GGGCTGTG >-	7:140501243G > A	16:30080955G > A	22:19960473T > C	22:20796725C > T	10:108589363T > C	22:21331358G > A	15:94901832G > A	3:142136034C > T
DNA change	c.1854 + 1G > A	c.769C > T	c.1214C > G	c.1129C > T		c.3303_3310del	c.829C > T	c.922G > A	c.2525A > G	c.1540G > A	c.695A > G	c.1256G > A	c.1292G > A	c.1384G > A
Amino acid change	—	p.Arg257Trp	p.Pro405Arg	p.Arg377Cys		p.Q1101fs	p.Pro277Ser	p.Val308Ile	p.Asp842Gly	p.Val514Met	p.Tyr232Cys	p.Arg419Gln	p.Arg431His	p.Ala462Thr
rs number	rs371704346	rs757250411	—	rs752385379		rs777383420	—	rs142759891	—	rs748490204	rs201223592	rs775926412	rs375821657	rs1223545973
MAF gnomAD	0.0002	0.00002	0	0.0006		0.000003	0	0.00005	0	0.00003	0.00008	0.00006	0.00002	0
MAF gnomad FIN	0	0	0	0.00009		0.0006	0	0.0002	0	0.00017	0.0005	0.0001	0	0
MAF obesity cohort	0.0054	0.0054	0.0054	0.0054		0.0054	0.0054	0.0054	0.0054	0.0054	0.0054	0.0054	0.0054	0.0054
CADD	34	26.3	28.9	23.8		33.0	27.1	23.3	22.4	33.0	27.0	25.6	24.3	27.9
MutationTaster2	Disease causing	Disease causing	Disease causing	Disease causing		NA	Disease causing	Disease causing	Disease causing	Disease causing	Disease causing	Disease causing	Disease causing	Disease causing
M-CAP	NA	Damaging	Damaging	Tolerated		NA	Damaging	Damaging	Damaging	Damaging	Damaging	Tolerated	Damaging	Tolerated
REVEL	NA	Benign	Pathogenic	Benign		NA	Pathogenic	Benign	Benign	Pathogenic	Benign	Benign	Benign	Benign
SIFT	NA	Damaging	Damaging	Tolerated		NA	Damaging	Tolerated	Damaging	Damaging	Damaging	Tolerated	Tolerated	Damaging
Polyphen2	NA	Probably Damaging	Probably Damaging	Benign		NA	Probably Damaging	Benign	Benign	Probably Damaging	Probably Damaging	Probably Damaging	Probably Damaging	Posssibly Damaging
VarSome ACMG Classification (criteria used)	VUS (PP3, BS1)	VUS (PM2, PP3)	VUS (PM2, PP3, BP1)	LB (BS1, BP1, BP4)		VUS (PVS1, PP3, BS1)	LP (PM2, PM1, PP2,PP3)	VUS (PM2, PP3)	VUS (PM2, BP1)	VUS (PVS1, PM2, PP3)	LB (PP3, BS1, BP1)	LB (BS1)	VUS/B (BP1)	VUS (PM2, PP3)

MAF gnomad FIN; minor allele frequency in gnomad Finnish population

NA; data not available

ACMG; American College of Medical Genetics and Genomics

VUS; Variant of uncertain significance

B; benign

LB; likely benign

LP; likely pathogenic

Table 2 presents the cytogenic location of the genes with the rare variants. Four of the genes (*ALDOA*, *KIF22, SEZ6L2* and *TAOK2*) were in the 16p11.2 region and three of the genes (*AIFM3, ARVCF, KLHL22*) were in the 22q11.21 region. We further evaluated the gnomAD constrained metrics for the genes that were involved in the rare deletions that we previously identified. Table 3 presents the predicted constraint metrics. Three of the genes (*BRAF*, *TAOK2* and *XRN1*) had a pLI = 1, indicating dosage sensitivity to loss-of-function variants.

**TABLE 2:** The cytogenic location for the 12 genes with identified rare variants.

Cytogenic location	Gene symbol	Gene	Clinical genomic database	Inheritance
16p11.2	*ALDOA*	Aldolase, fructose-bisphosphate A	Glycogen storage disease XII	AR
16p11.2	*KIF22*	Kinesin Family Member 22	Spondyloepimetaphyseal dysplasia with joint laxity, type 2	AD
16p11.2	*SEZ6L2*	Seizure Related 6 Homolog Like 2	—	—
16p11.2	*TAOK2*	TAO Kinase 2	—	—
22q11.21	*AIFM3*	Apoptosis inducing factor mitochondria associated 3	—	—
22q11.21	*ARVCF*	ARVCF Delta Catenin Family Member	—	—
22q11.21	*KLHL22*	Kelch Like Family Member 22	—	—
7q34	*BRAF*	B-Raf Proto-Oncogene, Serine/Threonine Kinase	Noonan syndrome 7, cardiocutaneous syndrome, LEOPARD syndrome 3	AD
1q21.1	*CHD1L*	Chromodomain helicase DNA Binding Protein 1 Like	—	—
15q26.2	*MCTP2*	Multiple C2 And Transmembrane Domain Containing 2	—	—
10q25.1	*SORCS1*	Sortilin Related VPS10 Domain Containing Receptor 1	—	—
3q23	*XRN1*	5′-3′ Exoribonuclease 1	—	—

AR; autosomal recessive, AD; autosomal dominant

**TABLE 3:** Predicted constrained metrics for the genes involved in deletions.

Cytogenic location	Gene symbol	pLI	LOF Z-score	LOF o/e	LOEUF	Missense Z-score	Missense o/e	%HI
16p11.2	*ALDOA*	0	2.3	0.4	0.8	0.3	1.0	11.3
16p11.2	*KIF22*	0	2.2	0.6	0.9	0.1	1.0	33.8
16p11.2	*SEZ6L2*	0.12	4.4	0.3	0.4	1.8	0.8	34.6
16p11.2	*TAOK2*	1	6.0	0.13	0.2	3.2	0.7	27.9
7q34	*BRAF*	1	5.9	0.1	0.2	3.7	0.5	9
1q21.1	*CHD1L*	0	−0.2	1.0	1.3	−0.6	1.1	45.4
3q23	*XRN1*	1	8.1	0.12	0.2	2.1	0.8	23.3

pLI; an estimate of the probablility of being loss-of-funtion intolerant. pLI closer to 1 indicates that the transcript cannot tolerate protein truncating variation.

LOF; loss of function, Z-score; A greater Z-score indicates more intolerane to the class of variation.

o/e; Observed over expected ratio of variants in the gene. When a gene has a low o/e, it is under strong selection for that class of variation.

LOEUF; loss-of-function observed/expected upper bound fraction.

%HI; haploinsufficiency rank. Genes with lower numbers are more likely to be dosage sensitive.

We also explored coding and splice variants with an allele frequency of <0.005. These variants were in nine genes; *ARVCF, C16orf54, C22orf39, ESF1, GNB1L, KLHL22, MCTP2, MVP* and *TRMT2A* (Supplementary Table S2). We did not find any potential pathogenic/likely pathogenic variants or candidate genes in this second analysis.

Furthermore, we evaluated the subjects in our study with the rare CNVs to look for additional rare variants in the specific genes corresponding to the regions of the deletion since a heterozygous deletion can also affect the function of a gene by unmasking a rare pathogenic variant on the other allele following recessive inheritance. No rare variants were found in these genes, thus excluding biallelic presence of damaging variants.

## Discussion

CNVs are causing several diseases. However, in many cases it is challenging to determine the pathogenicity and clinical impact of the CNVs and to pinpoint the potential contribution of specific genes to the phenotype. In addition, the clinical relevance of rare CNVs can be difficult to determine because of incomplete penetrance and variable phenotypic expression. We performed targeted exome sequencing to identify rare variants in 117 genes located in previously identified CNV regions in subjects with severe early-onset obesity (Pettersson et al., 2017) in search for potential candidate genes. We found rare heterozygous variants with allele frequencies <0.1% in twelve of the CNV genes.

The 16p11.2 deletion is the most common CNV linked to severe obesity. However, the mechanism by which the proximal 16p11.2 deletion causes the phenotype is poorly understood and none of the genes in this region have so far been identified as a solely causative gene for the complex phenotype associated with proximal 16p11.2 CNVs. The patients with 16p11.2 deletions often present with various neurodevelopmental and neurobehavioral conditions, including autism spectrum disorders (ASD) and speech and language problems (Zufferey et al., 2012; Chung et al., 2021). The link between obesity and neurodevelopmental disorders may be due to common genes involved in the neurodevelopment that affect pathways regulating energy balance. Recently, new approaches for fine-mapping the genes in 16p11.2 region have been developed using GWAS and biobank data. Vysotskiy et al. performed an association analysis between imputed gene expression levels and BMI and they found four genes in the 16p11.2 region (*TMEM219*, *SPN*, *TAOK2* and *INO80E*) associated with BMI (Vysotskiy et al., 2021). We identified rare heterozygous variants in four genes in the proximal 16p11.2 region; a frameshift variant in *TAOK2* and heterozygous missense variants in *SEZ6L2*, *ALDOA* and *KIF22.* These four genes are potential candidate genes for the phenotype in patients with 16p11.2 CNVs. *TAOK2* has a pLI of 1, suggesting rare loss-of-function variants may be deleterious (Lek et al., 2016). *TAOK2* has been implicated in neurodevelopmental disorders and *TAOK2* mutations found in patients with ASD showed impaired dendrite and synaptic development; the BMIs in the affected patients were not reported (Richter et al., 2019).TAOK2 heterozygous and knockout mice show gene brain morphological and behavioral abnormalities (Richter et al., 2019). *SEZ6L2* has also been proposed as a candidate gene for ASD because of high expression in the brain (Kumar R. A. et al., 2009; Konyukh et al., 2011). Subcutaneous adipose tissue expression of *ALDOA* has been correlated with weight regain after dietary intervention (Roumans et al., 2017). *ALDOA* encodes an enzyme involved in glycolysis and the upregulation of glucose intake may be related to the risk of weight regain (Roumans et al., 2017). Furthermore, analysis of human fetal gene expression data found that *KIF22* and *ALDOA* are significantly enriched in progenitors compared to post-mitotic cells making them candidates for having specific roles in neurogenesis in the developing human fetal cerebral cortex. It has been suggested that neurogenesis is disrupted in 16p11.2 CNVs (Morson et al., 2021). The participants in our study with rare variants in *ALDOA, KIF22, TAOK2* and *SEZ6L2* did not present with ASD or neurodevelopmental disorders (Table 1). However, the subjects with the missense variants in *KIF22* and *SEZ6L2* had learning difficulties. To our knowledge, no variants in these four genes have been reported in monogenic obesity. We consider the contribution of the identified rare variants to development of obesity unclear.

Two of the CNVs (16p11.2 deletion and 22q11.21 duplication) that we previously identified in our obese cohort include over 20 genes. In large CNVs, several genes within the locus may contribute to the phenotype. Disruption of multiple genes may be responsible for the complex phenotype seen in patients with 16p11.2 deletions, explaining the difficulty of identifying a single causative gene in the 16p11.2 region. Jensen et al. proposed that haploinsufficiency of certain genes may be altered by haploinsufficiency of other genes in the same CNV region and that these genes interact with each other via shared biological pathways (Jensen and Girirajan, 2019).

Interestingly, we identified heterozygous missense variants in *SORCS1* in two subjects. Another study investigating genetic variants in 72 individuals with obesity also found two rare missense *SORCS1* variants of unknown significance (Ginete et al., 2021). *SORCS1* is highly expressed in the central nervous system and encodes a vacuolar protein sorting 10 domain-containing receptor that binds neuropeptides. *SORSC1* has been associated with insulin secretion and diabetes risk (Goodarzi et al., 2007) and loss of both *Sorcs1* and *Sorcs3* in mutant mice caused increased adiposity and enhanced food intake, but not increased body weight (Subkhangulova et al., 2018). The participants in our study with the rare variants in *SORSC1* did not present with insulin resistance or diabetes. In each family the variants were inherited from a parent with overweight, but overweight/obesity was present also in family members without the variant and therefore the contribution of these variants to the phenotype remains uncertain. More studies are needed to investigate the molecular mechanisms of *SORCS1* and the possible contribution of this gene to early-onset obesity.

Another gene of interest is *XRN1* located in 3q23 region. *XRN1* is highly constrained for both loss-of-function and missense variants. This gene encodes 5′-3′ Exoribonuclease 1, which is involved in replication-dependent histone mRNA degradation. No *XRN1* variants in humans have been associated with obesity, but a recent study investigating forebrain-specific *Xrn1* knockout mice found that lack of Xrn1 in neurons led to obesity, hyperphagia, leptin resistance and hyperglycemia. The researchers proposed that Xrn1 in the hypothalamus is important for the mRNA degradation in regulating gene expression in the leptin signaling pathway (Takaoka et al., 2021). In our study, we identified a heterozygous missense variant in *XRN1* of uncertain significance in a participant with isolated obesity. Further studies are required to explore the clinical relevance of this variant and biological function of *XRN1*.

Our study was limited by the lack of a control group of normal-weight subjects. However, we compared the allele frequencies of the variants we found in this study with the Finnish population in gnomAD and SISu project, with genotype data of more than 10,000 Finns. Because of the methodological limitation of targeted exome sequencing we may have missed variants in non-coding parts of the selected genes and variants in novel genes that were not included in the panel but may contribute to obesity. We may also have missed more common variants potentially contributing to the phenotype. Other limitations of this study were unavailability of parental DNA samples in part of the cohort and the lack of functional evaluation of the genetic variants. Further studies are needed to explore the possible role of the identified variants in the development of obesity.

In conclusion, the genes and mechanisms mediating the obesity phenotype in the previously identified CNVs remains uncertain and more studies are needed to provide insight to the potential contribution of the rare CNVs and the specific genes to severe obesity.

## Data Availability

The datasets presented in this article are not readily available because Data cannot be shared publicly because the data consists sensitive patient data. More specifically the data consists of individual clinical data and individual genotypes for young children. Data are available from the Helsinki University Hospital’s Institutional Data Access/Ethics Committee for researchers who meet the criteria for access to confidential data. Requests to access the datasets should be directed to Outi Mäkitie MD, Ph.D., outi.makitie@helsinki.fi.
